# Prevalence of Vitamin B12 Deficiency Among Diabetic Patients Taking Metformin in Jordan

**DOI:** 10.7759/cureus.82672

**Published:** 2025-04-21

**Authors:** Sakher S Ja'anini, Malak S Ababneh

**Affiliations:** 1 General Practice, Al-Mahabba Hospital, Madaba, JOR; 2 General Practice, Ministry of Health-Jordan, Irbid, JOR

**Keywords:** diabetes mellitus, metformin, type 2 diabetes mellitus, vitamin b12, vitamin b12 deficiency

## Abstract

Background

Type 2 diabetes mellitus prevalence in Jordan is high and increasing, leading to an increase in the use of metformin, which is considered the first-line treatment. Metformin, which belongs to the biguanides family of anti-diabetic medication, is known for its low cost, bodyweight neutrality, effectiveness, and good safety profile. In addition, it showed that it improves body lipid profile, inflammatory markers, and decreases cardiovascular events. In spite of that, a reduction in vitamin B12 levels was reported as a possible long-term side effect of metformin. Vitamin B12 deficiency can result in macrocytic, megaloblastic anemia, it can also cause neuropathies, including subacute combined degeneration, neuropsychiatric symptoms, peripheral neuropathy, and optic neuropathy.

Aims

The current study aimed to identify the level of serum vitamin B12 among patients who use metformin. Additionally, the study aimed to explore associations between serum vitamin B12 levels and patients’ age, gender, duration, and dosage of metformin use, and concurrent medications. Moreover, the study summarizes participants’ socio-demographical and pharmacological data and presents the statistical analysis methods used to answer the research questions.

Methods

A cross-sectional study was conducted on a sample that included 435 patients suffering from type 2 diabetes mellitus at the internal medicine clinics at Al-Mahabba Hospital, Madaba, Jordan. After applying the inclusion and exclusion criteria that we have designed, information regarding their age, gender, metformin dosage, treatment duration, and other medications in use was precisely collected, followed by ordering serum vitamin B12 level and obtaining the results as soon as they were available. We diagnosed vitamin B12 deficiency depending on a cut-off value for serum vitamin B12 level of less than 200 pg/ml. The collected data were entered into an Microsoft Excel 16 (Microsoft Corp., Redmond, WA) sheet and then transferred to the statistical software, IBM SPSS Statistics for Windows, Version 28.0 (IBM Corp., Armonk, NY) to analyze the data.

Results

A total of 435 patients on metformin were participated, the majority of patients were male with sample mean age of 59 years, most of patients use metformin three times a day with 13.9 years of metformin duration of treatment, the serum vitamin B12 level was found to be 188.9 pg/ml which considers deficient with a prevalence of 66%. The patients’ age, metformin dose, and duration were negatively correlated with serum B12. The patients’ gender did not show an impact on vitamin B12 levels. The type of medications in use with metformin demonstrated a significant impact on B12 level, as evident by the post hoc test.

Conclusion

According to the findings, we concluded that patients who have used metformin are likely to be more vulnerable to vitamin B12 deficiency than those who have not used metformin. There is an obvious link between the patient's age, dosage, and length of metformin usage and the vitamin B12 deficiency. The patients’ gender did not show an impact on vitamin B12 levels. Also, other medications in use with metformin, such as insulin, vildagliptin, histamine 2 blockers, and proton pump inhibitors, have an impact on vitamin B12 levels.

## Introduction

Many studies have studied the association between metformin use and vitamin B12 deficiency, but up until now, there are no guidelines especially in Jordan requiring regular screening for vitamin B12 among metformin users. Here, the question of whether metformin is associated with vitamin b12 deficiency or not arises.

Metformin, which belongs to biguanides, is a very popular drug for treating diabetes mellitus type 2. It has considered as the first line of treatment in various guidelines including the European Association for the Study of Diabetes (EASD) and American Diabetes Association (ADA) [1]. This is due to its low cost, body weight neutrality, effectivity and good safety profile [1]. In addition, it showed that it improves body lipid profile, inflammatory markers and decreases cardiovascular events [1]. The risk of lactic acidosis due to the use of metformin is minimal [2]. Compared to, phenformin and buformin, which also belong to biguanides, and which were used earlier as drugs for hyperglycemia, they were drown from markets of most countries because they induce lactic acidosis [3]. 

Metformin has multiple mechanisms and sites of action, but they are still not fully understood [4]. Regarding to its glucose lowering effect it has shown to reduce liver glucose production directly and indirectly, increase gut glucose utilization, increase GIP-1 and alter the microbiome [4].

Vitamin B12, which is also called cyanocobalamin, is synthesized only by microorganisms, stored and transferred by animal products [5]. Vitamin B12 is very important in DNA synthesis and odd chain fatty acids metabolism by acting as a cofactor for methionine synthase (transfers CH3 group as methylcobalamin) and methylmalonic-CoA mutase [6]. Vitamin B12 deficiency can result in macrocytic, megaloblastic anemia [7]. In addition, it can cause neuropathies including subacute combined degeneration, neuropsychiatric symptoms, peripheral neuropathy and optic neuropathy [8]. Furthermore, vitamin b12 deficiency results in mildly elevated homocysteine levels, which increase the risk of thrombosis by causing endothelial cell damage [9].

Natural vitamin B12 is tied to proteins and, when consumed by humans, is liberated in the stomach and then bound to glucagon proteins (R-binders) [10]. R-binders are produced by salivary glands and the stomach, they protect vitamin B12 from denaturation in the stomach's strong acidic environment [10]. R-binders are digested in the duodenum, releasing free vitamin to attach directly to another glycoprotein termed intrinsic factor (IF), which travels through the bottom section of the small bowel and is absorbed by particular ileal receptors [10]. The binding of vitamin B12-IF to its binding receptors in the membrane of ileal cells was discovered to be highly reliant on calcium ion concentrations in the small bowel [10].

Vitamin B12 is stored in our bodies in higher quantities than any other vitamin: between 2000 g and 4000 g, the body may theoretically function for up to 10 years on a daily supply of 1 g; this is one of the reasons why the catastrophic consequences of vitamin B12 deficiency might take a long time to manifest [11]. The liver contains the most vitamin B12, followed by the kidney and spleen [11].

The importance of this research arises as the prevalence of diabetes mellitus type 2 in Jordan is high and is increasing [12], and the most of them are treated with metformin either as monotherapy or in combination, so it is very important to detect if metformin is associated with vitamin B12 deficiency or not. Strategies should be implemented to detect the association between metformin and vitamin B12 deficiency. In addition, to detect the prevalence of vitamin B12 deficiency among metformin users, in order to educate the health professionals if this association is present. This research aims to make metformin use safer to provide better health outcomes among its users by setting a general recommendation for early vitamin B12 deficiency detection.

## Materials and methods

Following the Ethical Guidelines of Clinical Research in Jordan and the approval from Al-Mahabba Hospital's ethics reviewing committee, a cross-sectional study involving 435 patients was conducted on the outpatients of the internal medicine clinics at Al-Mahabba Hospital, Madaba, Jordan. The study lasted for a duration of two and a half years.

Inclusion criteria

The study involves patients who are diagnosed with diabetes mellitus type two and are either on metformin as monotherapy or in combination with other antidiabetic medications, including those who also use histamine 2 blockers or proton pump inhibitors in addition to their antidiabetic medication.

Exclusion criteria

Many pathologies can cause vitamin B12 deficiency through affecting the digestion and absorption pathway of vitamin B12, including autoimmune gastritis, Crohn’s disease, celiac disease, alcohol cirrhosis, aging related atrophic gastritis, small intestinal bacterial overgrowth, HIV patients especially who are co-infected with tuberculosis and, exocrine pancreatic dysfunction [13-20]. Additionally, bariatric surgeries and ileal resection, particularly when the terminal ileum is involved, have been associated with an increased prevalence of vitamin B12 deficiency among patients, primarily due to the surgical interference with the digestion and absorption processes of this essential vitamin [21-22].

Furthermore, multiple drugs have the potential to cause vitamin B12 deficiency when they are used chronically, such as colchicine, chloramphenicol, and ethanol. Collectively, these drugs illustrate the complex interplay of various substances that can lead to impaired vitamin B12 absorption and utilization in the body [23]. Finally, there is a high prevalence of vitamin B12 deficiency among long-term vegans and vegetarians; this is related to the low B12 intake from natural food sources [24]. For those reasons, any patients who have these pathologies, have undergone these surgeries, are on one of these medications, on a vegan or vegetarian diet, or have recently received vitamin B12 injections have been excluded from the study to decrease the bias as much as possible.

Diagnosing vitamin B12 deficiency

Advanced vitamin B12 deficiency presents with macrocytic RBCs with hypersegmented neutrophils, glossitis, subacute combined degeneration of the spinal cord, low serum vitamin B12, high serum homocysteine (similar to folate deficiency), and high serum methylmalonic acid (unlike folate deficiency) [25]. Laboratory measurement of serum vitamin B12 level of less than 200 pg/mL (148 pmol/L) is consistent with vitamin B12 deficiency (specificity of 95-100%) [25]. We depended on the laboratory measurement of serum vitamin B12 level of less than 200 pg/mL to diagnose vitamin B12 deficiency in our study, due to its relatively lower cost, objectivity, and availability compared to the other tests and criteria. The tests were done in the hospital's laboratory using the Roche Cobas e 411 (Roche Diagnostics, Basel, Switzerland) machines.

Data collection

Patients who were diagnosed with type two diabetes mellitus and were following up at Al-Mahabba Hospital's internal medicine clinics were randomly picked and then filtered using our inclusion and exclusion criteria. Information regarding their age, gender, metformin dosage, treatment duration, medical and surgical history, their diet, and other medications in use was precisely collected by direct history taking during their follow-up visits and/or from their medical records. The patient's contact information, for example, mobile phone numbers, was obtained to get any other missing information. Any patient who had any missing information that was not reached was excluded from the study. Following that, serum vitamin B12 level was ordered on the individual level, and results collection was done as soon as they were available. The collected data were entered into an Microsoft Excel 16 (Microsoft Corp., Redmond, WA) sheet and then transferred to the statistical software, IBM SPSS Statistics for Windows, Version 28.0 (IBM Corp., Armonk, NY) to analyze the data. Standard data scanning and cleaning were performed using descriptive statistics. The frequency and percentage were applied for categorical data, while the mean ± SD was applied for scale data (age, serum vitamin B12, metformin dose, and duration). Additionally, the assumptions of normality, linearity, and homoscedasticity for Pearson correlation, while the assumptions of normality and homogeneity of variance for the independent t-test and one-way ANOVA were all satisfied.

## Results

Participants’ socio-demographic and pharmacological characteristics

A total of 435 type 2 diabetic patients were enrolled in the study. Among the 435 participants, 288 (66.2%) were male and 147 (33.8%) were female patients with a sample mean age of 59.2±12.4 (Table 1). Over three-quarters of patients use metformin three times a day at 333 (76.5%), while a minority of six (1.4%) used it once per day, with an average of 2334.3±403.4 mg of metformin consumed daily. Furthermore, the mean of serum vitamin B12 was found to be 188.9±69.8, which is less than 200 pg/ml and points to vitamin B12 deficiency among the studied sample, and measuring the prevalence, 66% of the metformin users have vitamin B12 deficiency. However, the duration of metformin use was 13.9±4.7 years, and finally, about one-third of patients were not going on any medication other than metformin

**Table 1:** Participants’ socio-demographic and pharmacological characteristics *1, *2, *3 indicates the frequency of the daily tablet dose

Variables	Categories	Frequency	Percentage	Mean±SD
Gender	Male	288	66.2	
	Female	147	33.8	
Age in years				59.2±12.4
Metformin dose/day	850 mg *1	6	1.4	
	850 mg *2	96	22.1	
	850 mg *3	333	76.5	
Metformin dose/mg				2334.3±403.4
Metformin duration/years				13.9±4.7
Serum vitamin B12 level				188.9±69.8
Medications with metformin	Non	144	33.1	
	Lansoprazole	95	21.8	
	Famotidine	51	11.7	
	Insulin	82	18.9	
	Vildagliptin	63	14.5	

Association between serum vitamin B12 and patients' socio-demographics and pharmacological data

Numerous statistical analysis techniques were used to answer the research question, the Pearson product moment correlation was used to explore correlation between serum vitamin B12 with patients’ age, metformin daily doses and duration of metformin treatment while an independent t-test and one-way ANOVA were performed for mean differences of vitamin B12 level based on patients’ gender and type of medications use respectively.

The results of Pearson product moment correlation show that the serum vitamin B12 was significantly negative correlated with patients’ age (r= -.264), metformin daily doses (r= -.311) and duration of metformin treatment (r= -.348) p<0.001 respectively (Table 2).

**Table 2:** Correlation analysis between serum vitamin B12 and patients characteristics

Variable	Age	Metformin daily doses	Duration of metformin treatment
Serum vitamin B12	-.264	-.311	-.348
p value	<0.001	<0.001	<0.001

Furthermore, serum vitamin B12 levels were investigated based on patients’ gender and type of medications. The results reveal that the mean of serum vitamin B12 was not statistically significantly different based on patients’ gender (p=0.098) while the one way ANOVA showed that serum vitamin B12 was significantly different based on types of medication (p<0.001) (Table 3). In addition, a least significant difference (LSD) post-hoc test was used for family-wise comparisons and the results have shown that patients ongoing on insulin and vildagliptin had a significantly higher serum vitamin B12 than those who did not take medication with metformin (p<0.001).

**Table 3:** Correlation analysis between serum vitamin B12 level and patient's characteristics ^t^Independent t-test, ^F^One way ANOVA

Variables	Category	N	Mean	SD	Test value	p value
Gender	Male	288	192.87	72.13	1.659^t^	0.098
	Female	147	181.16	64.58		
Medications with metformin	Non	144	170.80	59.16	6.600^F^	<0.001
	Lansoprazole	95	187.14	68.38		
	Famotidine	51	180.53	71.96		
	Insulin	82	209.80	71.50		
	Vildagliptin	63	212.63	78.32		

Moreover, the insulin and vildagliptin patients significantly reported a higher vitamin B12 serum than patients going on lansoprazole (p=.028 and p=.022 respectively) and similarly they demonstrated a higher vitamin B12 level than patients ongoing on famotidine (p=.016 and p=.013 respectively) (Table 4).

**Table 4:** LSD post-hoc test for family-wise comparisons *The mean difference is significant at the 0.05 level, **The mean difference is significant at the 0.001 level LSD: least significant difference

Medications with metformin (I)	Medications with metformin (J)	Mean difference (I-J)	Std. error	Sig.	95% Confidence interval	
					Lower bound	Upper bound
Non	Lansoprazole	-16.34	9.00	.070	-34.03	1.35
	Famotidine	-9.73	11.09	.381	-31.54	12.07
	Insulin	-39.00^**^	9.42	< .001>	-57.52	-20.49
	Vildagliptin	-41.83^**^	10.28	< .001>	-62.05	-21.62
Lansoprazole	Famotidine	6.61	11.82	.576	-16.62	29.84
	Insulin	-22.66^*^	10.26	.028	-42.84	-2.50
	Vildagliptin	-25.49^*^	11.06	.022	-47.24	-3.76
Famotidine	Insulin	-29.27^*^	12.14	.016	-53.14	-5.41
	Vildagliptin	-32.11^*^	12.82	.013	-57.31	-6.90
Insulin	Vildagliptin	-2.83	11.41	.804	-25.25	19.59

Results summary

A total of 435 patients on metformin were participated, the majority of patients were male with sample mean age of 59 years, most of patients use metformin three times a day with 13.9 years of metformin duration of treatment, the serum vitamin B12 level found to be 188.9 pg/ml which considers deficient.

The patients’ age, metformin dose and duration were negatively correlated with serum vitamin B12 level. The patient's gender did not show an impact on vitamin B12 level. The type of medications demonstrated a significant impact on B12 level as evident by post hoc test, the patient who were on insulin and vildagliptin reported a higher vitamin B12 level than who on other medication types. 

## Discussion

Based on our results, we demonstrated that the patient’s age, daily metformin dosage, and treatment duration, were the most consistent risk factors for vitamin B12 deficiency. As all of them are negatively correlated with b12 levels. To the best of our knowledge, this is the first large-scale study that was specifically designed to investigate the prevalence and contributing factors for vitamin B12 deficiency confined to a Jordanian population with type 2 diabetes treated with metformin.

As in western countries, metformin treatment with lifestyle modification is recommended as a first-line treatment for type 2 diabetes mellitus in Jordan, and subsequently other antidiabetic agents are added in the aim of controlling hyperglycemia. Metformin is the most frequently prescribed oral antidiabetic agent in Jordan.

In this study, vitamin B12 deficiency was present in (66%) of patients using metformin. The reported prevalence of vitamin B12 deficiency related to metformin use varies according to the study population. Data from the research done by El-Khateeb et al., measuring the prevalence of vitamin B12 deficiency among Jordanian population concludes that 30.1% of Jordanians have vitamin B12 deficiency using the cut off value of serum vitamin B12 level of less than 200 pg/ml [26]. Comparing our results with this research’s results, we will find that the attributable risk for metformin in the sitting of vitamin B12 deficiency is 35.9%, and the relative risk is 2.19 indicting the presence of correlation between metformin use and vitamin B12 deficiency. 

Our study showed a clear relationship between the patient’s age, dosage and length of metformin use and vitamin B12 deficiency in patients with type 2 diabetes. The patients’ gender did not show an impact on vitamin B12 level. Also, patients ongoing on insulin and vildagliptin had a significantly higher serum vitamin B12 than those who did not take medication other than metformin. Furthermore, the insulin and vildagliptin patients reported a significantly higher vitamin B12 serum than patients going on lansoprazole; they demonstrated a higher vitamin B12 level than patients ongoing on famotidine. Surprisingly, metformin monotherapy has been associated with the lowest vitamin B12 level mean.

Despite the numerous articles linking metformin to vitamin B12 deficiency, little attention has been paid to the mechanism by which this impact occurs [10]. The lack of focus on the mechanism might be owing to the ongoing, contentious discussion regarding the impact itself [10]. Although there is a lack of literature, we found different mechanisms such as the study by Hassan and Alhagry that mentions that the most basic hypothesis is that changes in small intestinal motility induced by metformin contribute to bacterial overgrowth and consequent vitamin B12 consumption [10]. Although, in a controlled trial [27], the mechanisms under serious consideration include those based on metformin-induced biochemical changes in calcium status, which are necessary for the absorption of vitamin B12-intrisic factor by ileal cell receptors. Metformin blocks calcium channels in the distal ileum, which change the membrane potential and enable absorption of the vitamin B12-intrinsic factor (B12-IF) complex [28]. Metformin's hydrophobic tail attaches to the cell membrane's hydrocarbon core, providing a net positive charge that repels calcium, which is required for delivering B12 across the ileal-lumen interface [28].

Vitamin B12 deficiency is clinically important because it is a reversible cause of bone marrow failure and demyelinating nerve disease. Neurologic damage, a possible consequence of metformin-induced vitamin B12 deficiency, can present as peripheral neuropathy and may be mistaken for diabetic neuropathy in patients on metformin treatment. As vitamin B12-associated neuropathy is a treatable and reversible condition, early detection and treatment of vitamin B12 deficiency is clinically important in patients with diabetes using metformin.

Limitations

The findings of this study are derived from a single hospital that serves a relatively lower socio-economical population when compared to other hospitals in Jordan. Thus, the findings may not be generalized to the broader national context, highlighting the need to collect data from multiple hospitals or clinics to gain better understanding of the relation between metformin and vitamin B12 deficiency and how other factors such as age, gender, dosage, duration, and other medication in use can affect it.

## Conclusions

According to the findings of the study involving 435 patients, we found that patients who have used metformin are likely to be more vulnerable to vitamin B12 deficiency than those who have not used metformin. There is an obvious link between the patient's age, dosage and length of metformin usage and the vitamin B12 deficiency, as all of these factors are negatively correlated with vitamin B12 level. The patients’ gender did not show an impact on vitamin B12 level. Additionally, patients ongoing on insulin and vildagliptin had a significantly higher serum vitamin B12 than those who did not take medication other than metformin. Furthermore, the insulin and vildagliptin patients reported a significantly higher vitamin B12 serum than patients going on lansoprazole; they demonstrated a higher vitamin B12 level than patients ongoing on famotidine. Surprisingly metformin monotherapy has been associated with the lowest vitamin B12 level mean. Screening for vitamin B12 deficiency among metformin users is highly advised due to its crucial role in the body. Dietary modification and vitamin B12 supplementation are also advised among people who use metformin due to the risk of vitamin B12 deficiency.
